# Reverse Adipofascial Radial Forearm Flap Surgery for Soft-Tissue Reconstruction of Hand Defects

**Published:** 2016-12-23

**Authors:** Osman Akdag, Mehtap Karamese, Muhammed NebilSelimoglu, Ahmet Akatekin, Malik Abacı, Mustafa Sutcu, Zekeriya Tosun

**Affiliations:** ^a^Department of Plastic Reconstructive and Aesthetic Surgery, Selcuk University, Konya, Turkey; ^b^Department of Plastic Reconstructive and Aesthetic Surgery, Medipol University, Istanbul, Turkey

**Keywords:** reverse adipofascial radial forearm flap, hand, soft-tissue defect, radial forearm flap, donor-site morbidity

## Abstract

**Objective:** The reverse radial forearm flap has been used for soft-tissue hand defect surgical procedures worldwide. One of the major drawbacks of this flap, however, is donor site morbidity, as the donor site is closed with a skin graft. Problems with skin graft donor areas include adhesion, contracture, and wound-healing complications. In this study, only the adipofascial component of a reverse radial forearm flap was used to prevent these problems; in addition, a skin graft was applied over the flap instead of over the donor site. **Methods:** Between January 2011 and December 2013, a total of 13 hand defects were reconstructed with a reverse adipofascial radial forearm flap. Patients were evaluated for functional results using total active motion criteria and disability of the arm, shoulder, and hand scores, operation time, hospitalization time, and patient satisfaction. **Results:** All flaps and grafts placed on flaps survived completely and donor sites healed without complications. The total active motion criteria and the disability of the arm, shoulder, and hand score demonstrated that the functional outcomes were successful. Patient satisfaction scores using the visual analog scale had a mean of 88.3 (SD = 2.95) mm. Operation time for the flap surgery was 126.1 (SD = 21.80) minutes, and patients were discharged at an average of 6.3 (SD = 1.44) days. **Conclusion:** Use of an adipofascial component in reverse radial forearm flap surgery is appropriate for reducing problems with donor site skin grafts. Patients' functional outcomes denoted that the reverse adipofascial radial forearm flap is a reliable and effective method to cover soft-tissue defects of the hand.

**Level of Evidence: IV**

Hand injury may cause the tendons, cartilage, bones, nerves, and joints to become exposed. To maintain hand function and to protect a hand's vital structures, good coverage must be achieved as part of the repair process. Many types of flaps for dorsal and palmar defects of the hand have been described in the literature.^1^ The flap preference for hand defects is often related to a surgeon's experience, the injury site, and the vascular form of the patient's hand and forearm. Ideal flap coverage must provide restoration of hand function and minimize donor site morbidity.^2^

A radial forearm flap takes the form of a pedicled or free flap for reconstruction of soft-tissue defects. A reverse radial forearm flap is distally based on retrograde flow through the ulnar artery and palmar arches after ligation of the proximal radial artery.^3^ The venous drainage is carried out through the concomitant veins of the radial artery.^4^

Reverse radial forearm flap construction is not a microvascular technique and is used successfully and effectively for soft-tissue hand defects when deep vital structures such as joints, bones, or tendons are exposed. A reverse pedicled flap with a strong blood supply offers a safe, simple, and effective 1-stage procedure. ^5^ Donor and recipient sites are located in the same operative field, which may be an advantage for some surgeons. However, an unpleasing cosmetic problem of donor site closure is one of the drawbacks of this procedure.^6^ We prefer to use an adipofascial component of the reverse radial flap and apply a skin graft over the adipofascial flap instead of the donor site. This modification may provide better results aesthetically and functionally because the forearm region is protected from the disadvantages of a skin graft.

This article aims to present technical details, advantages, and disadvantages of using a reverse adipofascial radial forearm flap (RARFF) by reviewing the results of 13 patients whose soft-tissue hand defects were reconstructed using this procedure.

## MATERIALS AND METHODS

### Patients

Thirteen patients (9 males, 4 females) had soft-tissue defects of the hand and were referred to our medical institute between January 2011 and December 2013; patients age ranged from 21 to 67 (mean = 33.84, SD = 11.48) years. Patients’ demographic characteristics are summarized in Table 1. The defects are described as follows: all defects were on the dorsum of the hand; 5 patients had tendon injury and 4 had bone fracture (Table 1). This procedure was used in patients who had no comorbid disease, and their vascular competencies were confirmed by an Allen's test and a portable Doppler ultrasound scan.^3^ Patients who had comorbid disease or radial artery dominancy were excluded. This study was conducted with approval from the Local Ethical Committee.

### Operative techniques

**Figure 1:**
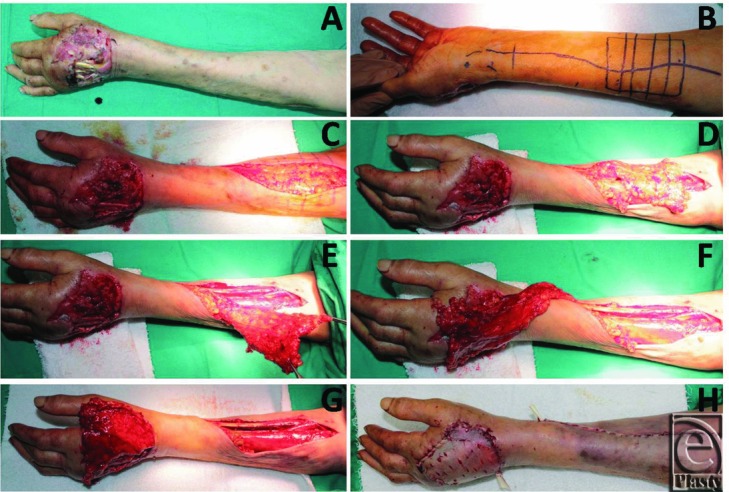
(a) The defect of the dorsum of the hand in a 67 years old woman. (b) Flap dimensions are designed according to the size of the defect. (c) Skin incision. (d) Exposure of adipofascial tissue. (e) Dissection of the vascular pedicle. (f) Dissection precedes the radial styloid. (g) Flap adaptation on the defect area. (h) Graft adaptation over the flap.

The dorsal hand defects that had exposed bones, tendons, nerves, or joints were closed with RARFFs. General anesthesia was used for all patients. Realignment and stabilization of metacarpal bone fractures and tendon repair were performed before the flap design. The RARFF was designed in the forearm region according to the size of defect area (Figs 1*a* and 1*b*). This surgical procedure was chosen for dorsal hand defects that had bone and tendon exposure. The skin was incised with an S-shaped incision (Fig 1*c*). The incised skin was undermined and separated from the underlying adipofascial tissue (Fig 1*d*). It was important that sufficiently thick skin be left at the donor area. Adipofascial flap borders that included the forearm fascia were incised cautiously, and radial artery perforators and adipofascial tissue were protected. The radial artery and concomitant veins were dissected and ligated in the proximal forearm (Fig 1*e*). Extreme care was taken to preserve the lateral antebrachial cutaneous nerve.^3^ Flap dissection was carried out from the proximal to the distal area and to the pivot point, which is located 1 to 2 cm above the radial styloid (Fig 1*f*). The RARFF was transposed to the defect using a subcutaneous tunnel, if needed, or was transposed directly to the defect and sutured to the defect area (Fig 1*g*). A skin graft was applied over the flap (Fig 1*h*). The forearm skin that was left at the donor site was sutured directly, and a lightly compressive dressing was applied. A suction drain was used for 1 day.

### Evaluation criteria

All patients were followed up for 6 to 20 months, and postoperative evaluation was undertaken at 6 months using the total active motion (TAM) score of the metacarpophalangeal joint; disability of the arm, shoulder, and hand (DASH) score; patient satisfaction score; operation time; and time of hospitalization.

The TAM score was used for finger function. A score between 260° and 270° was considered excellent; 259° to 221°, good; 200° to 220°, normal; 180° to 199°, fair; and less than 180°, poor.^2^

The DASH score was used to evaluate the functional outcomes of the patients. The questionnaire comprises 30 items and ranges from 30 to 150 points, with 30 indicating full activity and 150 indicating limitations.^7^ Data were transformed using the following formula: [(sum of *n* responses/*n*) − 1] × 25; this made it easy to compare with another measures scaled on a 0- to 100-point scale.

To evaluate patient satisfaction, a 100-mm visual analog scale (VAS) was used. The VAS ranged from 0 mm for *unsatisfied* to 100 mm for *very satisfied*.

Operation and hospitalization durations were noted for all patients.

## RESULTS

We performed 13 RARFF procedures (Table 1). The flaps ranged from 55 to 100 mm in length and from 45 to 80 mm in width. No patient needed tendon or bone grafts. All donor sites were closed primarily. All flaps survived, and all donor areas, both adipofascial flap and skin graft, healed with no complications. There were no patient complaints of cold intolerance, pain, or numbness in the forearm or hand.

Functional outcomes of the finger were excellent and good in 11 and 2 patients, respectively, according to the TAM criteria. The DASH questionnaire results demonstrated that postoperative functions were excellent and symptoms were minor. The average DASH score was 9.9 (SD = 4.18).

**Figure 2:**
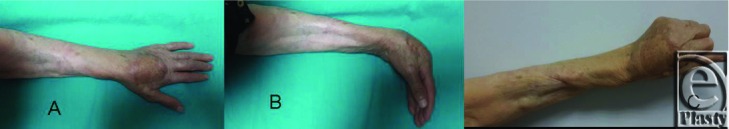
(a-c) Late postoperative results of the patient whose surgical details are explained in Figure 1.

**Figure 3:**
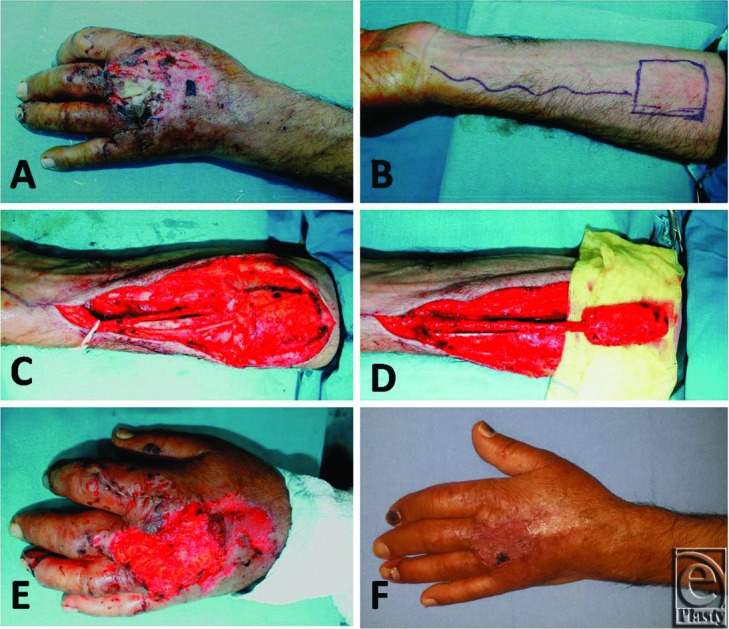
(a) The defect of the dorsum of the hand in a 42 years old man. (b) Design of the flap. (c, d) Elevation of the flap. (e) Flap adaptation. (f) Postoperative view at 6 months.

**Figure 4:**
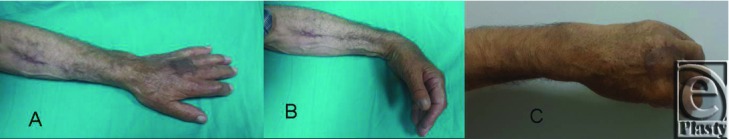
(a-c) Late postoperative results of the patient whose surgical details are explained in Figure 3.

**Figure 5:**
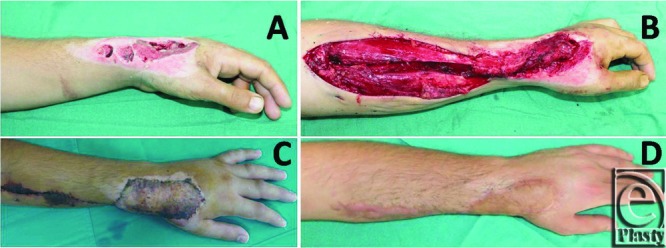
(a) Dorsal hand defect. (b) Flap transposition. (c) Early postoperative result. (d) Late postoperative result.

Patients reported an 88.3 (SD = 2.95) mm satisfaction score in terms of the donor site and the healing process (Figs 2-5).

Operation time for flap surgical procedures was 126 (SD = 1.80) minutes. Patients were discharged 5 to 9 days after surgery (mean = 6.3 days, SD = 1.4 days).

## DISCUSSION

**Figure 6:**
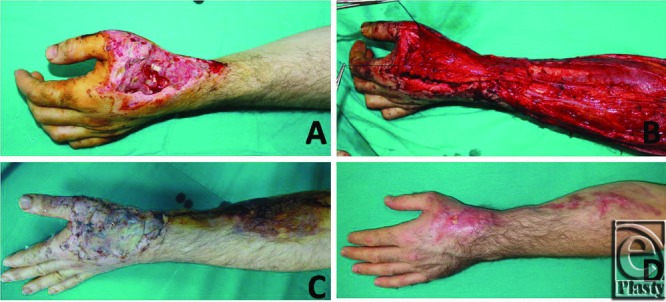
(a) Dorsal hand defect in a 31 years old man. (b) Flap transposition. (c) Early postoperative result. (d) Late postoperative result.

**Figure 7:**
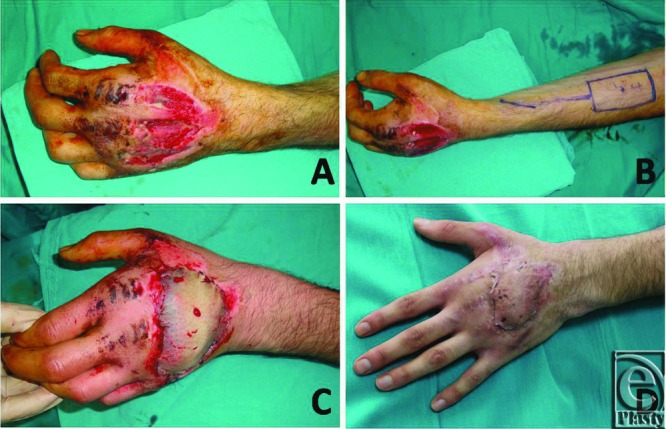
(a) Dorsal hand defect in a 28 years old man. (b) Flap transposition. (c) Early postoperative result. (d) Late postoperative result.

In this study, soft-tissue hand defects were reconstructed successfully in 13 patients using an RARFF, which used only the adipofascial component of a traditional reverse radial forearm flap. No skin grafts were used for donor sites, and all donor sites were closed primarily. The reconstruction option for soft-tissue hand defects lies between using a distant flap pedicled on the groin and a microsurgical flap using microvascular techniques or local perforator flaps. Early reconstruction and mobilization are advocated for reconstruction of the upper extremities to improve the whole functional outcome, preserve the remaining tissue, and prevent desiccation and infection; thus, 1-stage procedures are preferred over multistage ones, including the groin, inferior hypogastric, and abdominal flaps.^8^ Free flaps provide excellent coverage for soft-tissue defects but are restricted by donor site morbidity and their feasibility when there is a need for microsurgery (Figs 6 and 7).

The reverse radial forearm flap is a 1-stage procedure used for the reconstruction of soft-tissue hand defects worldwide. The donor site in a traditional reverse radial forearm flap procedure is closed with a skin graft.^9^ The limitation of this flap procedure is the sacrifice of a major artery that may jeopardize hand viability. The radial forearm perforator flap, which preserves the radial artery and prevents the need to sacrifice the radial artery, is described for covering moderate-sized hand and wrist defects.^8^ It cannot be stated that the RARFF is superior to the radial forearm perforator flap. However, in this article, we tried to minimize the disadvantages of a traditional reverse radial forearm flap arising from a skin graft to the donor area. One of the major disadvantages of the forearm flap is its significant donor site morbidity.^9^ The most frequently reported donor site problems are delayed healing of skin graft, adhesions, edema, contracture, and sensory changes. To minimize donor site complications, the use of tissue expansion and artificial dermis is advocated.^6,9-11^ Morbidity of a donor site must be taken into consideration when planning hand reconstruction.

Although there is a high incidence of free radial forearm flap use, new approaches to donor site management have been encouraging. Khan et al^12^ harvested a free adipofascial radial forearm flap with limited skin incisions and the aid of lighted retractors. The authors recommended harvesting the adipofascial flap to avoid donor site morbidity; however, their technique is demanding and prolongs the operating time.^12^ The adipofascial component of a reverse radial forearm flap can be obtained easily via an S-shaped incision. According to Akyürek et al,^5^ an oblique radial forearm flap for closure of the donor site helps skin tension lines. In the Ahn et al^6^ report, the transverse radial artery forearm flap for donor site closure was created through V-Y advancement of a fasciocutaneous flap. In addition, closure of the donor site has been carried out with a V-Y flap in cases where the defect width in the longitudinal axis of the forearm does not exceed 4 cm.^13^ Hsieh et al^14^ introduced a perforator-based bilobed flap to cover donor defects of a radial forearm flap. All of these modifications have been made to minimize donor site complications. We prefer to use a skin graft above the flap on the dorsum of the hand for minimizing the donor site skin defect. After obtaining soft-tissue support for a hand defect, a skin graft on the RARFF causes less damage than that in the forearm region, where a traditional reverse radial forearm flap is grafted.

An RARFF can be used safely with a skin graft over the flap instead of a donor site. Our patients reported their satisfaction with a mean patient satisfaction score of 89 (SD = 3.2) mm; the score was accepted as satisfied and meaningful. Another issue is that when a skin graft is used on a donor site, adhesion may result. Adhesion between skin grafts and muscle bellies may limit forearm function. If a skin graft is placed over a flap, primary closure can be achieved for the original forearm skin; however, the loss of subcutaneous volume at the donor site inevitably causes a slight contour defect. The risk of adhesion is minimal. Moreover an S-shaped incision is more cosmetically pleasing than a skin graft closure. This procedure minimizes donor site morbidity and significantly improves the aesthetic result.

In anatomical studies, it was shown that radial artery perforators supply the forearm skin.^15^ In our operations, because of the surgical procedure, the radial artery was sacrificed and the skin was supplied by the ulnar artery perforators after elevation of the adipofascial radial artery flap. We believe that the “dense subdermal vascular plexus”^15^ is involved in this process. After surgery, the skin looks purplish, but it resolves within a few days.

The results of TAM and DASH questionnaires have encouraged us to continue using this modification of a radial forearm flap. However, this surgical procedure needs to be compared with the traditional reverse radial forearm flap procedure in randomized controlled trials.

RARFFs may ensure confident coverage of the hand with a strong blood supply and primary closure of the donor site, thereby eliminating complications from the conventional radial forearm flap donor site.

## Figures and Tables

**Table 1:** Clinical data of reverse adipofascial radial forearm flaps*

				Bone					
			Flap size,	fracture/					
			mm (length	tendon	Operation	Hospitalization			
Patients	Sex	Age, y	× width)	injury	time, min	time, d	TAM	DASH	PSS
1	M	28	55 × 45	−/−	130	7	Excellent	15	83
2	M	30	60 × 50	+/−	120	5	Excellent	8.3	90
3	M	31	80 × 50	−/−	130	9	Excellent	16	84
4	F	67	100 × 80	+/+	120	9	Good	16	92
5	M	21	80 × 70	−/+	180	6	Excellent	16	90
6	F	33	90 × 70	+/+	160	7	Good	8.3	90
7	F	34	65 × 55	+/+	100	7	Excellent	5.8	90
8	F	27	65 × 45	−/−	120	7	Excellent	5.8	85
9	M	32	60 × 40	−/−	130	6	Excellent	5	90
10	M	23	70 × 50	−/−	110	5	Excellent	8.3	90
11	M	42	80 × 70	−/+	110	5	Excellent	8.3	90
12	M	39	70 × 60	−/−	120	5	Excellent	8.3	85
13	M	33	75 × 65	−/−	110	5	Excellent	8.3	90

*TAM indicates total active motion; DASH, disability of the arm, shoulder, and hand; and PSS, patient satisfaction score.
